# Vital Pulp Therapy in Teeth with Symptomatic Irreversible Pulpitis: A Systematic Review

**DOI:** 10.3290/j.ohpd.b5718325

**Published:** 2024-08-29

**Authors:** Arwa S. Bafail

**Affiliations:** a Assistant Professor, Restorative Dental Science Department, College of Dentistry, Taibah University, Madinah Munawara, Saudi Arabia. Data collection, wrote the manuscript, reviewed the literature, read and approved the final manuscript.

**Keywords:** inflammation, irreversible, symptomatic, vital pulp therapy

## Abstract

**Purpose:**

Nonsurgical root canal therapy (NSRCT) is indicated for management of permanent teeth diagnosed with symptomatic irreversible pulpitis. However, recent research has suggested that vital pulp therapy (VPT) may be a less invasive option in these cases. The purpose of this systematic review was to evaluate the outcomes of VPT, using hydraulic calcium silicate cements (HCSCs) including complete and partial pulpotomies in permanent posterior teeth with symptomatic irreversible pulpitis.

**Materials and Methods:**

The PRISMA recommendations were adhered to. The search approach used electronic databases from PubMed, EMBASE, the Cochrane Library, and grey literature. The Newcastle-Ottawa Scale, ROBINS-I, and Cochrane Collaboration Risk of Bias tools were used to evaluate the quality of the selected studies.

**Results:**

The initial database search turned up 142 papers, of which 3 prospective cohort studies and 9 randomised controlled trials were selected for analysis. For three, seven, and two articles, the risk of bias was rated as “high” or “serious,” “fair,” and “low,” respectively. The success rates for VPT using HCSCs typically ranged from 78% to 90% one to five years following VPT. The results of the VPT and NSRCT were equivalent at one and five years, according to two articles. Although the intra-operative pulp assessment is essential for VPT treatments, most studies did not provide a thorough account of this process or the time required to achieve haemostasis. Three studies reported sample sizes that were < 23 teeth. The 12 studies that were analysed revealed successful VPT procedures using HCSCs in permanent posterior teeth that had symptomatic irreversible pulpitis, with radiographic success rates ranging from 81% to 90%. Two articles claimed that the results of VPT and root canal therapy were equivalent.

**Conclusion:**

When considering VPT as an alternative to NSRCT, appropriate case selection and outcome criteria must be created. This data highlights the need for additional studies contrasting the longer-term effects of different treatment regimens.

An inflammation of the dental pulp tissue brought on by detrimental stimuli, such as bacterial invasion of the tooth structure, is referred to as pulpitis.^21^ It usually progresses from a stage that is reversible, if the stimulus is eliminated, to an irreversible stage, if it is not. The clinical diagnosis of pulpal and periapical tissues is established^49^ based on clinical signs and symptoms, caries extension, responses to pulp and periapical testing, and the radiographic presentation of the periapical tissues at the root apex. Severe pain pre-operatively, either elicited or spontaneously. Carious pulp exposure has traditionally been viewed as signs of irreversible pulpitis. However, in teeth showing these symptoms, the inflammatory changes are often limited to the coronal pulp adjacent to the carious lesions^6–10,45,46^.While the majority of the pulp tissue remains viable and uninflamed.^36^ The outdated categorization of irreversible pulpitis has thus been called into question due to the growing understanding that removing the severely inflamed pulp tissue may permit the retention of the remaining uninflamed pulp, making the pulpitis reversible.^36,49^ This growing knowledge has an impact on the methods used to treat teeth with irreversible pulpitis.^37,49^ Nonsurgical root canal treatment (NSRCT) is the conventional treatment of choice for permanent teeth that have been diagnosed with irreversible pulpitis. The main steps and objectives of the NSRCT procedure access to qualified healthcare professionals who can administer it.^38^ In those circumstances, the irreparable pulpitis- diagnosed teeth may be removed, endangering function and quality of life.^47^ Based on radiographic and clinical criteria, 92% to 98% of the teeth treated with NSRCT that satisfies the specified process goals should continue to function normally years after treatment.^17,32,39^ On the other hand, high prevalence values of apical periodontitis (34 to 68%) and insufficient root fillings (56 to 86%) have been reported by various cross-sectional studies globally evaluating root canal filled teeth. These findings suggest that often neither the expected outcomes nor the accepted process goals are achieved.^18,19,33,24^ Vital pulp therapy (VPT), which generally includes partial pulpotomy (PP) and full pulpotomy (FP) procedures, is seen as an alternative to NSRCT for teeth with pulp inflammation.^20^ VPT has long been regarded as the only option for treating deciduous teeth with inflamed pulp and permanent immature teeth with reversible or irreversible pulpitis.^2,6,15,30^ It is congruent with modern notions of minimally invasive dentistry.^13^ “The development of hydraulic calcium silicate cements (HCSCs), such as Biodentine, calcium enriched mixture cement (CEM), and mineral trioxide aggregate (MTA) (Septodont; Saint-Maur-des-Fossés, France)”, which successfully seal the exposed pulp tissue and promote pulp healing, has strengthened VPT over the past 20 years.^14,31,48^ Permanent mature teeth with symptomatic irreversible pulpitis has been treated by FP^6-11,25,35,42,43^.This finding suggests that even those teeth which were once thought to be beyond pulpal repair can be predictably treated by removing the inflamed and affected tissue, maintaining the vitality of the remaining pulp and periapical health.^6-11,16,25,26,35,42-44,45,46^ The main goal of this systematic review was to evaluate the most recent research on the effectiveness of VPT employing HCSCs for permanent adult posterior teeth that had been diagnosed with symptomatic irreversible pulpitis. “The question pursued in this research was, after treatment with VPT in permanent mature posterior teeth diagnosed with symptomatic irreversible pulpitis, what is the radiographic, clinical, or total prognosis? One of the study’s secondary goals was a comparison of the results between the VPT and NSRCT”.

## MATERIALS AND METHODS

PRISMA recommendations^28^ were adhered to. Electronic databases from the Cochrane Library, EMBASE, and PubMed were used for the main search.^28^ It was restricted to works published between the years of 2000 and 2021 (PubMed/Medline, Embase, and Cochrane: from January 1, 2000, to March 1, 2021). For “pulpitis”, “irreversible”, “pulpotomy,” and “vital pulp therapy,” free phrases and medical descriptors (such as MeSH terms) were employed, joined by the Boolean operator “AND” (e.g., PubMed search strategy: “Irreversible” AND “pulpitis” AND “Pulpotomy”[Mesh] OR “pulpotomy”). Grey literature searches (using Open Grey and ClinicalTrials.gov), cross-searches in pertinent journals for this topic, and references listed in the papers found and systematic reviews were included in the primary search.

### Data Extraction and Analysis

Using EndNote software (Clarivate Analytics; Philadelphia, PA, USA), articles were assessed for inclusion in three stages, as follows:

- A preliminary search and the removal of duplicates.
- The electronic search results were independently evaluated by title by reviewer. The abstract was examined for eligibility when a title seemed pertinent.
- The whole content of the article was examined when the title and abstract were deemed pertinent. Minor revisions were made after a unique data extraction form was established and pilot tested on two studies for feasibility and thoroughness.

### Recall Rate and Treatment Outcomes

The recall rate was obtained by dividing the total number of treated teeth by the number of teeth found in follow-up exams. Success, defined as the absence of clinical indicators (sinus tract, swelling, oedema, or redness), symptoms (pain, sensitivity to percussion, or palpation), and a normal radiographic appearance of the periapical tissues, was the main outcome of interest. “The success rate for each study was the number of teeth recorded as “success” divided by the total number of treated teeth”.

### Quality Assessment

Two approaches were used to assess the risk of bias in Cochrane Collaborations. The RoB 2 and ROBINS-I tools were used in randomised controlled trials and non-randomised investigations, respectively (https://www.riskofbias.info). Cohort studies further used the Newcastle-Ottawa Scale.

## RESULTS

### Selected Studies

After the electronic search in PubMed, EMBASE, the Cochrane library was complete and duplicate entries were eliminated, the abstracts of 117 articles were examined, leading to the deletion of 102 articles (Tables 1–3). Following a full text review, three more articles were eliminated because the diagnosis of pulpitis did not meet the criteria for inclusion^22^ or because authors were not available to provide additional clarification of the published data on the outcome for teeth with irreversible pulpitis^11,44^ (Table 4). There were no further papers that fit the inclusion criteria found by a hand search or a review of the grey literature. The 12 articles included in this systematic review are shown in Table 5. Eight of the articles^6,7,25,35,42,43,45,46^evaluated the results of PP or FP performed using various materials. At three follow-up intervals, three articles^8-10^ reported the results of FP or NSRCT. The results of FP conducted with various materials over the course of three follow-up periods were discussed in two studies.^6,7^ Another publication^26^ discussed the results of FP and NSRCT conducted in various roots of the same tooth compared to NSRCT performed in all of the roots of treated teeth. One subset of patients who had FP treatment utilising CEM cement were documented,^6,8^ with follow-up periods of two years^7,9^ and five years.^7,8^ The primary author did not respond to a question asking for more information regarding the potential duplication of this group. Immature permanent molars were occasionally included in samples in three studies,^35,43,46^but only the data for mature permanent teeth were examined in those studies.

**Table 1:** Articles excluded after title and abstract screening using PubMed/Medline Database

Articles	Reason for exclusion
Eren et al, 2018; Kérourédan et al, 2017; Brignardello-Petersen, 2017; Bane et al, 2016; Asgary and Eghbal, 2010; Nyerere et al, 2006	Only clinical outcome available
Asgary and Ramazani 2018; Asgary et al, 2017; Ashraf et al, 2017; Soni 2016; Asgary et al, 2016; Asgary and Kemal 2015; Solomon et al, 2015; Asgary 2011	Case report
Wang et al, 2020; Sabbagh et al, 2016; Harandi et al, 2013	Case report Immature permanent teeth
Peng et al, 2015	Chinese language Immature permanent teeth
Chen et al, 2020; Ghaderi et al, 2020; Memarpour et al, 2016; Parisay et al, 2015; Whaterhouse et al, 2002	Primary teeth
Mousavi et al, 2016; Mente et al, 2016; Chueh and Chiang 2010; Eghbal et al, 2009; Sharma et al, 2020	Histological and biological study
Jalali et al, 2015; Dunlop et al, 2013; Elsharraww and Elbaghdady 2007; Bagheri et al, 2019	Unrelated to the topic
Simon et al, 2013; Tan et al, 2020	No symptoms of irreversible pulpitis
Orhan et al, 2010	Indirect pulp therapy
Asgary and Eghbal 2010	Retracted article
Yazdani et al, 2014	Health technology assessment
Asgary and Ehsani 2009	Case series
Zafar et al, 2020; Sadaf 2020	Systematic review/review
George 2020; Bakhurji 2020	Opinion article


**Table 2:** Articles excluded using the EMBASE database

Articles	Reason for exclusion
Zanini et al, 2017; Rechenberg et al, 2016; Lin et al, 2020	Inflammatory mediators and histological studies
Yu et al, 2020; Chompu-Inwai et al, 2018	Unrelated to the topic
Chen et al, 2020	Primary molars
Sabeti et al, 2021	Animal model
Wang 2020; Ramezani et al, 2020	Case report and immature tooth
Kusumvalli et al, 2019	Absence of signs and symptoms of irreversible pulpitis


**Table 3:** Articles excluded using the Cochrane database

Articles or main ID of clinical trials	Reasons for exclusion
NCT03956199 NCT00748280 NCT04573374 NCT04397315 NCT04308863 NCT03186690 NCT04243733 NCT03916900 IRCT20151226025695N3 NCT03735069	Clinical trials without results reported
RCT2013030512708N1 NCT03168620 NCT04599244 Jalali et al. 2015 Bane et al. 2016 Kérourédan et al. 2017 Elsharrawy and Elbaghdady 2007 IRCT201110137790N1 IRCT20181021041405N1 IRCT2017101036699N1 CTRI/2019/09/021443 CTRI/2019/09/021443 RBR.5j25nm ISRCTN14290358	Clinical trials/articles unrelated to the topic
Eren et al, 2018 ISRCTN14290358 Asgary and Eghbal, 2010	Clinical outcome/pain relief
McDougal, 2004	Intermediate restoration
Asgary and Eghbal, 2010	Retracted

**Table 4:** Articles excluded after full-text reading

Author(s)	Title	Reason for exclusion
Asgary et al, 2018	Treatment outcomes of 4 vital pulp therapies in mature molars	The outcome was unclear. Specific outcome for teeth with irreversible pulpitis not discernible.
Taha et al, 2017	Assessment of mineral trioxide aggregate pulpotomy in mature permanent teeth with carious exposures	The outcome was unclear. Specific outcome for teeth with irreversible pulpitis not discernible from reversible pulpitis. Authors were not available to provide additional clarification of the published data.
Galani et al, 2017	Comparative evaluation of postoperative pain and success rate after pulpotomy and root canal treatment in cariously exposed mature permanent molars: a randomized controlled trial	Diagnosis of pulpitis was not consistent with inclusions criteria.

**Table 5:** Articles included in this systematic review

Authors, year	Title	Design
Asgary et al, 2013	One-year results of vital pulp therapy in permanent molars with irreversible pulpitis: an ongoing multicenter, randomized, non-inferiority clinical trial	Randomised controlled trial
Asgary et al, 2014	Two-year results of vital pulp therapy in permanent molars with irreversible pulpitis: an ongoing multicenter, randomized clinical trial	Randomised controlled trial
Asgary et al, 2015	Five-year results of vital pulp therapy in permanent molars with irreversible pulpitis: a non-inferiority multicenter, randomized clinical trial	Randomised controlled trial
Asgary and Eghbal, 2013	Treatment outcomes of pulpotomy in permanent molars with irreversible pulpitis using biomaterials: a multi-center, randomized controlled trial	Randomised controlled trial
Asgary et al, 2017	Long-term outcomes of pulpotomy in permanent teeth with irreversible pulpitis: a multicenter, randomized controlled trial	Randomised controlled trial
Kumar et al, 2016,	Comparative evaluation of platelet-rich fibrin, mineral trioxide aggregate, and calcium hydroxide as pulpotomy agents in permanent molars with irreversible pulpitis: A randomized controlled trial	Randomised controlled trial
Qudeimat et al, 2017	Mineral trioxide aggregate pulpotomy for permanent molars with clinical signs indicative of irreversible pulpitis: a preliminary study	Randomised controlled trial
Taha and Abdulkhader, 2018^38^	Full pulpotomy with Biodentine in symptomatic young permanent teeth with carious exposure	Prospective cohort study
Taha and Abdelkhader, 2018^39^	Outcome of full pulpotomy using Biodentine in adult patients with symptoms indicative of irreversible pulpitis	Prospective cohort study
Taha and Khazali, 2017	Partial pulpotomy in mature permanent teeth with clinical signs indicative of irreversible pulpitis: A randomized clinical trial	Randomised controlled trial
Uesrichai et al, 2019	Partial pulpotomy with two bioactive cements in permanent teeth of 6-to-18-year-old patients with signs and symptoms indicative of irreversible pulpitis: a non-inferiority randomised controlled trial	Randomised controlled trial
Koli et al, 2021	Combination of nonsurgical endodontic and vital pulp therapy for management of mature permanent mandibular molar teeth with symptomatic irreversible pulpitis and apical periodontitis	Randomised controlled trial

### Quality Assessment

Nine of the articles reviewed were randomised controlled trials. The final risk of bias for these articles was calculated by the Cochrane Collaboration Rob 2 tool (Tables 6 and 7), and were determined to be ‘low’ for one,^46^ ‘fair’ for seven,^10,26,45^ and ‘high’ for one.^25^ The risk of bias was rated as ‘low’ for two of the three examined prospective cohort studies using the Cochrane Collaboration ROBINS I instrument (Table 8) and as ‘serious’ for one.^35^ The same three studies received four, five, and six^35,42,43^ out of a possible nine stars when evaluated using the Newcastle-Ottawa Scale. The results of the two papers which had ‘high’^25^ or ‘serious’^35^ risk of bias were considered secondary to those of the remaining 10 articles.

**Table 6:** Risk-of-bias in randomized controlled trials based on the Cochrane Collaboration RoB 2 tool

	Asgary et al, 2013	Asgary et al, 2014	Asgary et al, 2015	Asgary and Eghbal 2013	Asgary et al, 2017	Kumar et al, 2016	Taha and Khazali 2017	Uesrichai et al, 2019	Koli et al, 2021
1. Random sequence generation	low	low	low	uncertain	uncertain	low	low	low	low
2. Allocation concealment	low	low	low	low	low	low	low	low	low
3. Blinding of participants and personnel	uncertain	uncertain	uncertain	low	low	uncertain	low	low	uncertain
4. Blinding of outcome assessment	low	low	low	low	low	low	low	low	low
5. Incomplete outcome data	uncertain	uncertain	uncertain	uncertain	uncertain	uncertain	low	low	low
6. Selective reporting	low	low	low	low	low	low	low	low	low
7. Other sources of bias									
7.1. Group imbalance	low	low	low	low	low	low	low	low	low
7.2. Sample size	low	low	low	low	low	low	uncertain	low	uncertain
7.3. Clinician bias	low	low	low	low	low	high	low	low	low
8. Final risk of bias	fair	fair	fair	fair	fair	high	fair	low	fair

**Table 7:** Risk of bias assessment justification for randomised clinical trials

Author, year	The risk of bias justification
Asgary et al, 2013	Blinding of participants and personnel – uncertain Different protocols for each treatment Incomplete outcome data – uncertain Number of teeth excluded after intraoperative assessment of pulp necrosis or haemostasis not achieved is not presented
Asgary et al, 2014	Blinding of participants and personnel – uncertain Different protocols for each treatment Incomplete outcome data – uncertain Number of teeth excluded after intraoperative assessment of pulp necrosis or haemostasis not achieved is not presented
Asgary et al, 2015	Blinding of participants and personnel – uncertain Different protocols for each treatment Incomplete outcome data – uncertain Number of teeth excluded after intraoperative assessment of pulp necrosis or haemostasis not achieved is not presented Loss to follow-up greater than 20%
Asgary and Egbhal, 2013	Random sequence generation – uncertain Information is missing Incomplete outcome data – uncertain ‘Number of teeth excluded after intraoperative assessment of pulp necrosis or haemostasis not achieved’ is not presented. Requests to the authors to clarify this point were not answered.
Asgary et al, 2017	Random sequence generation – uncertain Information not provided Incomplete outcome data – uncertain Number of teeth excluded after intraoperative assessment of pulp necrosis or haemostasis not achieved is not presented. Requests to the authors to clarify this point were not answered.
Kumar et al, 2016	Blinding of participants and personnel – uncertain Flow of the treatment protocol described was not in agreement with randomization presented in the manuscript (PRF preparation started before the beginning of pulpotomy treatment) Incomplete outcome data – uncertain Loss to follow-up greater than 20% Clinician bias – high Non-calibration or blinding of radiographic evaluators
Taha and Khazali, 2017	Sample size – uncertain No statistical calculation was presented to establish the sample size.
Koli et al, 2021	Blinding of participants and personnel – uncertain Tested materials and techniques are quite different and not possible to mask Sample size – uncertain No statistical calculation was presented to establish the sample size.

**Table 8:** Risk-of-bias in prospective cohort studies based on Cochrane Collaboration ROBINS-I tool

	Qudeimat et al, 2017	Taha and Abdulkhader, 2018^38^	Taha and Abdelkhader, 2018^39^
1. Bias due to confounding	low	low	low
2. Bias in selection of participants into the study	serious*	low	low
3. Bias in classification of interventions	low	low	low
4. Bias due to deviations from intended interventions	low	low	low
5. Bias due to missing data	serious**	low	low
6. Bias in measurement of outcomes	low	low	low
7. Bias in selection of the reported result	low	low	low
Overall bias	serious	low	low

*Diagnosis included (i) intermittent or spontaneous, sharp or dull, localized, diffuse, or referred pain; (ii) rapid exposure to dramatic temperature changes elicited heightened and prolonged episodes of pain even after the thermal stimulus has been removed; and (iii) no clinical symptoms but pulpal bleeding produced by caries excavation. A specific end-point of the follow-up is not presented.

### Outcome

Results for one year,^6,10,25,26,42,43^ two years,^7,9,45^ and three to five years^7,8,35,46^ were reported. At the one-year follow-up, high radiographic success rates of 92% to 98% and high clinical success rates of 92% to 100% were reported for FP utilising either MTA, CEM cement, or Biodentine.^6,10,42,43^ For PP employing ProRoot MTA white (Dentsply Maillefer; Ballaigues, Switzerland), a reduced total success rate of 83% was noted.^45^ PP employing calcium hydroxide was reported to have a 55% overall success rate,^45^ although this method was not targeted this review. Notably, in one study,^25^ the ProRoot MTA-based FP clinical success rate was 83% and the radiographic success rate was 53%; these were much lower than those reported in the other investigations. However, the study’s lower than expected recall rate and substantial risk of bias undermined these statistics. Clinical and radiographic success rates were 98% and 86%, respectively, at two years for FP using CEM cement.^7,9^ In contrast to calcium hydroxide, which had a success rate of only 43%, ProRoot MTA had an overall success rate of 85%. The present review, however, did not cover this procedure/operation.^45^ At 3 to 5 years, PP in which ProRoot MTA and Biodentine were employed, respectively reported clinical success rates of 85% and 90%, radiographic success rates of 92% and 100%, and total success rates of 85% and 90%.^46^ Radiographic success rates for FP with CEM cement or ProRoot MTA were 78% and 85%, respectively, whereas the clinical success rate was 98%.^7^ The overall success rates of FP utilising CEM cement and ProRoot MTA have also been reported to be 78%^5^ and 100%,^35^ respectively. Nonetheless, the reliability of these results is questionable, due to one study’s poor recall rate and the other’s “serious” risk of bias. At three follow-up intervals, the results of FP utilising CEM cement and NSRCT were correlated.^5-7^ At one year^10^ and two years,^9^ both treatment techniques had high clinical success rates of above 97%; however, NSRCT reported lower success rate when compared to FP. There was no significant difference in the overall success rates of 78% and 75% at five years^8^ did not statistically significantly differ between FP and NSRCT, although a low recall rate may weaken the reliability of the data. In molar roots with vital pulps, FP done with MTA while NSRCT was carried out in the different roots had a 93% one-year overall success rate, compared to 90% for NSRCT conducted in both molar roots.^26^

## DISCUSSION

Similar diagnostic standards for irreversible pulpitis were utilized in all the trials analyzed, including prolonged spontaneous or induced pain. Both asymptomatic and symptomatic irreversible pulpitis are referred to as “clinical diagnoses based on subjective and objective findings indicating that the vital inflamed pulp is incapable of healing” by the American Association of Endodontists.^4^This idea is supported by the evidence from the current systematic review, which supports the findings of other systematic reviews^3,16,21,27^ and indicates that symptomatic irreversible pulpitis in permanent teeth can be successfully treated in a more conservative treatment options such as VPT procedures, including FP or PP. This systematic review was conducted using well-recognized best practices. The search was conducted in accordance with PRISMA criteria^28^ and was restricted to recent articles from 2000 to 2023. Since varied pulpal diagnoses, immature teeth, and VPT utilizing other materials were not included in this study, it was limited to permanent posterior teeth diagnosed with symptomatic irreversible pulpitis treated by PP, FP, or a combination of NSRCT and FP. The effectiveness of the chosen studies was evaluated using the Newcastle-Ottawa Scale and two Cochrane risk of bias assessment techniques. Contrary to two earlier systematic reviews,^3,16^ treatment recommendations for particular procedures was not aimed in this review. So, the level of evidence of the reviewed studies was not taken into account. This was primarily due to the insufficient sample sizes (ranging from 13 to 23 teeth) in three of the studies,^21,35,43^ which made it difficult. Additionally, it was believed that a meta-analysis of the studies’ findings was inappropriate due to the heterogeneity^51^ that would cast doubt on the interpretations of the findings. Adult posterior teeth with symptomatic irreversible pulpitis were examined in this study over a number of follow-up intervals. One year,^6,10,42,43^ two years,^7,9,45^ and up to five years^7,8^ following treatment, the results of both procedures carried out using HCSCs remained largely constant, with overall success rates in the range of 78% to 90%.^7,45,46^ Low recall rates^8^ and a significant risk of bias^35^ indicated by the assessment with the Cochrane Collaboration ROBINS I instrument and the Newcastle-Ottawa Scale may weaken the validity of the stated lower and greater success rates.

The successful results of VPT in adult permanent teeth supported earlier systematic evaluations.^3,16,27^ The histological studies evaluating reparative dentin bridge formation beneath dressing material and adjacent healthy pulp tissue following pulpotomy in teeth with irreversible pulpitis^12,14,37^ also seemed to fit well with them. The results were similar to those results of immature permanent teeth,^2,30^ indicating that mature pulps might recover from inflammatory alterations just as easily. The studies that were evaluated covered the FP and PP methods of pulpotomy.^7-10,35,42,43,45,46^ Although it has been stated that FP might eliminate inflammatory tissue more reliably,^2^ the same results were obtained for FP and PP, with the overall success rate exceeding 81%, indicating that both techniques can be regarded as predictable when performed accurately.

The results of FP and PP carried out with calcium hydroxide were recorded in two studies,^25,45^ along with the results of the same procedures carried out using HCSCs materials, although they were beyond the scope of this review. In one PP study,^45^ calcium hydroxide performed worse than MTA, validating earlier claims that calcium hydroxide performed worse than HCSCs materials in VPT tests.^31,38,48^ The other study’s^25^ ‘high’ risk of bias could call into question the validity of the equivalent FP results reported for MTA and calcium hydroxide. To date, little data exists comparing VPT to NSRCT. In a recent randomized controlled trial, the one-year overall success rates for FP with MTA in one root and NSRCT in the other root of the same tooth, and for NSRCT in both roots, were reported to be 93% and 90%, respectively.^26^ Another randomized controlled experiment was conducted with three follow-up periods to assess the outcomes.^8–10^ For FP and NSRCT the overall comparable five-year success rates of 78% and 75%, respectively, therefore, considered as alternative treatments for permanent mature teeth with symptomatic incurable pulpitis,^8^ although differences in success have been reported radiography after one and two years.^9,10^ A thorough evaluation of the amount of pulp tissue destruction using clinical and radiographic methods is necessary before considering a permanent tooth as a candidate for VPT.^50^ Confirmed pulpal condition should be evaluated intraoperatively by assessing pulpal bleeding, tissue colour, and consistency.^29^ Lack of pulpal bleeding following pulp exposure indicated that the coronal pulp may be infected or necrotic, contraindicating a VPT procedure.^37^ In one study,^42^ the pulps of 7/64 (11%) of the teeth diagnosed with irreversible pulpitis were intraoperatively found to be partially (3 teeth) or fully (4 teeth) necrotic, indicating that overestimation of the pulp’s suitability for VPT procedures can occur as a result of incorrect preoperative assessment.

This study serves as a good example of the importance of intra-operative pulp assessment. Importantly, the majority of the studies^6-10,25,35^ that were evaluated have not adequately reported the performance of the intra-operative pulp assessment step, despite the fact that it is necessary in VPT procedures. A pre-operative radiographic evaluation could potentially be unclear. On the one hand, even when the pulp is necrotic, periapical abnormalities may not be evident.^41^ On the other hand, as seen in several of the reviewed investigations,^6,10,35,42^ inflammed pulps that are in fact reparable may accompany periapical findings, previously thought to be a pathognomonic marker of pulp necrosis.

Therefore, the pulp’s suitability for VPT procedures may be underestimated in the context of pre-operative radiography indications. It has proven difficult to find a link between pulpal bleeding duration and VPT surgery results. The time required for hemostasis was not mentioned in a number of the evaluated papers.^6-10,25^ The remaining six articles estimated hemostasis periods of 2 to 3 min,^45^ 4 to 6 min,^43^ 8 to 10 min,^46^ or even 25 min.^35^ Therefore, the trials that were analyzed do not support the recommendation that pulpotomy^37^ should not be performed if bleeding does not stop after two minutes. Examples of local anesthetics with varying vasoconstrictor concentrations are 2% lidocaine with 1:80,000 epinephrine,^6-10,35,43,45^ 2% lidocaine with 100,000 epinephrine,^26^ and 4% articaine with 1:100,000 epinephrine.^42,46^ The manner in which pulp status is determined intraoperatively may be directly impacted by the anaesthetic’s composition, as it may have an effect on hemostasis. Although the perfect hemostasis strategy is still being sought, numerous techniques have been used, including compression with a dry cotton pellet, the use of saline solution, different amounts of sodium hypochlorite, hemostatic agents, chlorhexidine, and corticosteroid paste.^50^ It is also unknown how the patient’s age affects the prognosis. Many of the studies that were evaluated exclusively involved young patients, ranging in age from 9 to 17 years^35,43,46^ to 35 years,^25,26^ supporting the idea that young patients’ pulps have a greater capacity for recovery. The results of other trials,^6-8,10,42^ which included a larger age range of 9 to 69 years, did not indicate that the patient’s age affected the outcome of VPT.

As is customary, clinical and radiographic parameters were combined to evaluate the effectiveness of VPT in the trials under evaluation. The clinical success rate was marginally higher than the radiographic success,^6,7,9,10,35,42,43^ with one exception.^46^ In addition to probing depth, restoration integrity, discolouration, and movement, clinical effectiveness was predominantly determined by the absence of patient-reported complaints in various investigations.^26,43,45,46^ In one study, a favorable reaction to the cold test was necessary.^45^ Importantly, pulp disease can become necrotic even in the absence of symptoms,^37^ and pulp sensitivity tests may lose their validity due to deep restorations and the deposition of tertiary or reparative dentin.^51^ Furthermore, pulp sensitivity testing on pulpotomised teeth produces erroneous findings.^11^ A typical sign of radiographic success is the lack of anomalies in the periapical bone,^16,21,27^ which are interpreted and difficult to discern on periapical radiographs in the initial stages of tissue disintegration.^1^ Examination of dentinal bridge formation beneath the pulp capping material may not be a good indicator of treatment success because of insufficient mineralisation,^29,46^ overlapping roots, and restorative materials.^51^ In fact, none of the studies included here considered the establishment of a dentinal bridge. Consequently, both the clinical and periapical disease processes are momentarily slowed down after VPT treatment. Despite the positive results of VPT in permanent teeth with irreversible pulpitis that have been documented in this evaluation and a prior one,^16^ case selection, global targets and outcome criteria need to be developed for VPT when considered as a substitute for NSRCT.^51^ “Studies from different geographical regions and clinical settings are necessary to verify the comprehensive applicability of VPT on adult permanent teeth with irreversible symptoms, because the outcome of VPT depends on a number of procedural factors, including the anesthetic drugs used, strict disinfection and bleeding control, the technique of dead tissue removal,^29^ the type of permanent restoration,^50^ dressing materials,^45^ and the use of magnification.^37^ ”

## CONCLUSIONS

In the twelve papers that made up this systematic review, full and partial pulpotomies using hydraulic calcium silicate cements were successfully performed on permanent, adult posterior teeth that showed signs of irreversible pulpitis. According to two articles, the outcomes of root canal therapy and full pulpotomy were comparable. Small sample sizes, inconsistent indications and outcome criteria, and a lack of data on intra-operative pulp assessment to evaluate its status all undermined the validity of the results. Despite its limitations, this evidence provided ethical justification for future RCTs or large-scale pragmatic studies aimed at comparing the longer-term effects of these alternative therapy methods. Both modalities differ in possibly accessibility to care, cost and the degree of invasiveness. Therefore, carefully planned future studies are necessary to strengthen the evidence base that clinicians can use to counsel patients about their options for treating permanent mature teeth with irreversible pulpitis.
